# New Assignment of Mass Values and Uncertainties to NIST Working Standards

**DOI:** 10.6028/jres.095.008

**Published:** 1990

**Authors:** Richard S. Davis

**Affiliations:** National Institute of Standards and Technology, Gaithersburg, MD 20899

**Keywords:** calibration, international standards, kilogram, mass, national standards, SI, standards

## Abstract

For some time it had been suspected that values assigned to NIST working standards of mass were some 0.17 mg/kg larger than mass values based on artifacts representing mass in the International System of Units (SI). This relatively small offset, now confirmed, has had minimal scientific or technological significance. The discrepancy was removed on January 1, 1990. We document the history of the discrepancy, the studies which allow its removal, and the methods in place to limit its effect and prevent its recurrence. For routine calibrations, we believe that our working standards now have a long-term stability of 0.033 mg/kg (3*σ*) with respect to the national prototype kilograms of the United States. We provisionally admit an additional uncertainty of 0.09 mg/kg (3*σ*), systematic to all NIST mass measurements, which represents the possible offset of our primary standards from standards maintained by the Bureau International des Poids et Mesures (BIPM). This systematic uncertainty may be significantly reduced after analysis of results from the 3rd verification of national prototype kilograms, which is now underway.

## 1. Introduction

The kilogram (kg) is one of the seven base units which form the foundation of the Système International d’Unités or International System of Units, abbreviated SI. Used world wide to express the results of physical measurements, the SI specifies that the kilogram is the unit of mass and that the mass of the International Prototype Kilogram exactly equals 1 kg. The International Prototype referred to in the definition is a cylinder made of an alloy of platinum and iridium and stored at the International Bureau of Weights and Measures (BIPM) in France. The kilogram is thus the only remaining base unit of the SI to rely on an artifact for its definition.

When the SI was established, replicas of the International Prototype were manufactured by the BIPM for use as national prototype kilograms. At long intervals, the national prototypes are returned to the BIPM where their assigned mass is verified by measurements directly traceable to the International Prototype [1]. It was intended by the founders of the SI that the national prototype kilograms would be the primary mass standards within each country. There are, however, several practical difficulties with this scheme. The following discusses the reasons for these difficulties and the steps we have taken to overcome them.

In order for the kilogram unit to be useful, methods must exist to measure multiples and submultiples of 1-kg standards. These methods, when successful, rely on good equipment and sound experimental practice. In addition to these, a calibration service requires rigorous tests to maintain statistical control of the measurement process. At NIST, statistical rigor was introduced in the 1960s through the pioneering work of Pontius and Cameron [2]. Present methods are simply refinements of the system which they established.

The uncertainty of a 1-kg standard, expressed as a dimensionless ratio, propagates directly to mass values of multiples and submultiples derived from the standard. For example, if a kilogram standard has a relative uncertainty of 1 ppm^1^, all multiples and submultiples derived from the standard will have an uncertainty component of 1 ppm propagated from the standard. In the field of precision measurement, uncertainty is usually reported at an estimated level of 1 standard deviation. All uncertainties are combined by the root-sum-square (RSS) method according to guidelines recommended by the International Committee for Weights and Measures (CIPM) [3]. In NIST calibration reports, on the other hand, uncertainties are estimated at a level of 3 standard deviations. Furthermore, any uncertainty deemed “systematic” to a series of measurements is added directly to the “random” uncertainties, which are combined by RSS. However, in the rest of this paper, we follow the CIPM recommendations unless otherwise noted.

In addition to the SI, the United States recognizes the U.S. Customary System of units for legal metrology. In this system, the avoirdupois pound (lb) is the unit of mass. It is, by definition, exactly equal to 0.45359237 kg.

## 2. History of NIST Mass Standards Before 1980

### 2.1 Primary Mass Standards of Platinum-Indium

Kilograms K20 and K4 are the two national prototypes of the United States. Kilogram K20 has historically been considered the primary U.S. kilogram standard with K4 being relegated to use as a “check standard.” The history of these two artifacts through 1985 has already been documented in a previous report [1]. One important question which remained open in [1] is whether the mass values assigned by BIPM to their working standards have been consistent with the SI definition of mass. The cause for concern was that the embodiment of the SI definition, the International Prototype Kilogram, had not been used since 1946. This situation has changed within the past year as BIPM embarked on only the third calibration of national prototype kilograms since 1889. Preliminary results obtained by BIPM as a part of the 3rd verification confirm the long-term stability of their working standards to within required limits [4].

### 2.2 Secondary Mass Standards

Platinum-iridium alloy (approximate density 21,500 kg·m^−3^) is too expensive a material for widespread use. At present, stable alloys of non-magnetic stainless steel (approximate density 8,000 kg·m^−3^) are usually specified for use as secondary standards. Before such alloys were available, practical standards were typically made of plated brass (approximate density 8,400 kg·m^−3^). The densities of these alloys assume importance because mass metrology is almost always performed in the ambient air (density ca. 1.2 kg·m^−3^) using balances which are, in essence, force or torque transducers. The effect of air buoyancy thus becomes a confounding influence which must be removed by correction.

The size of the necessary buoyancy correction relative to the mass of interest is given by:

$$\left(1-\rho_{a}/\rho_{s}\right)/\left(1-\rho_{a}/\rho_{x}\right)-1\approx \rho_{a}\left(1/\rho_{x}-1/\rho_{s}\right),$$ (1)

where

*ρ*_a_ = ambient air density
*ρ*_s_ = density of the known standard
*ρ_x_* = density of the unknown secondary standard.

Equation (1) makes clear that, when comparing weights of nearly equal density, the importance of the correction is relatively small. Buoyancy corrections are typically 10 ppm between alloys of stainless steel and brass; corrections of less than 5 ppm are typical for comparisons between various alloys of non-magnetic stainless steel. (Specifications for the highest quality analytical weights limit the alloy density to within a narrow range in order to ensure that buoyancy corrections between nominally equal weights will be small.)

By contrast, the buoyancy correction between (i) primary standards of platinum-iridium alloy and (ii) secondary standards of brass or stainless steel typically ranges from 87–97 ppm. In our laboratory, the densities of secondary kilogram standards are determined by hydrostatic weighing. The density of ambient air is now determined from the CIPM-1981 equation-of-state for moist air [5]. The latter requires knowledge of ambient temperature, barometric pressure, relative humidity, and carbondioxide level. A discussion of the accuracy which can be expected from buoyancy corrections in our laboratory is given in [1].

The above considerations dictate that calibrations carried out by NIST on a routine basis be performed with secondary standards having a density near to that of the unknown weight.

#### 2.2.1 N_1_ and N_2_

Two weights, designated N_1_ and N_2_, have served as NIST secondary standards of mass since 1965. The weights were fabricated in 1948 of a nickel-chromium alloy having a nominal density of 8,340 kg·m^−3^, which is close to that of the brass weights which were then in common use. These weights were given an initial calibration in terms of a platinum-iridium prototype (K4) in 1948. They were recalibrated against both K20 and K4 in 1958. The newer calibration gave mass values which were systematically higher by about 0.06 mg/kg. There is no indication in the existing records what, if any, uncertainty was assigned to either calibration. When, in 1965, N_1_ and N_2_ were placed in service as secondary mass standards, the mass assigned to them was based on selected data from the 1958 series of measurements. Presumably, this decision was made because the 1958 measurements were performed by remote control on a two-pan, Rueprecht balance having a standard deviation below 0.02 mg. By 1965, this device had been replaced by a single-pan balance which was much more convenient to use but which had an inferior standard deviation of about 0.15 mg. Further, remote weighing was not possible on the single-pan balance.

Based on the 1958 measurements, the mass of N_1_ and N_2_ taken together was calculated to be:

R=2kg−10.059mg.

The difference in mass between N_1_ and N_2_ was calculated by pooling a large amount of data:

C=−19.476mg.

These two numbers, *R* and *C*, fix the individual values of each kilogram. The uncertainty in *C* is largely statistical in nature. It depends almost entirely on the standard deviation of the balance used to compare the mass of N_1_ with N_2_. Thus its uncertainty could be rigorously assigned. In addition, the significance of any measured change in C could also be determined.

The uncertainty of *R* was much more problematic. The statistical component of this uncertainty resulting from the balance used in the measurements may, of course, be calculated. There are at least two additional components which increase the uncertainty of *R* (but not of *C*):

1. The uncertainty in the accepted mass of K20 with respect to the International Prototype Kilogram.
2. The accuracy of the correction for air buoyancy between the platinum-iridium and the nichrome kilograms.

Rather than base an estimate of these uncertainties on what was considered insufficient metrological data, calibration reports prior to January 1, 1990 state:

“It is assumed that the present ‘accepted values’ of the two NIST standards at the 1 kilogram level, designated N_1_ and N_2_, are without error. Estimates of the uncertainty of the accepted values of the NIST standards relative to the International Prototype Kilogram can be provided on request. However, these estimates have no real meaning in either national or international comparison. This is because of the lack of sufficient data to provide a realistic estimate of the uncertainty in the values assigned to the prototype kilograms K20 and K4, particularly in regard to long term, or between-run variability. Changes in the accepted values for the NIST standards at the kilogram level, as and when they occur, will be reported in the scientific papers of the Bureau and will be given wide distribution…”

Except for the change in name of the institution, the above wording had been in place at least since 1967. The reports of that time (and well beyond) also referenced a technical note entitled “The Accepted Values of the NBS Standards at the 1 kg Level and Associated Uncertainty Estimates,” to be published at a future date. Unfortunately, this note was never produced. Section 3 of the present paper may therefore be regarded as fulfilling a promise of long standing.

In looking over calibration documentation extending back 25 years, it seems that the original intention was to reserve N_1_ and N_2_ for calibration of other working standards of similar density. These working standards would be used in routine calibration work and thereby would spare N_1_ and N_2_ from excessive wear. But the calibration of working standards of 1 kg could only be done on the single-pan balance mentioned above. Thus working standards would be assigned an uncertainty which was large relative to the precision of commercially available balances unless the calibration were based on the average of many measurements. But the latter strategy would no longer spare N_1_ and N_2_ from excessive use.

Faced with this problem, N_1_ and N_2_ began to be used as working standards themselves in routine calibrations. They were never cleaned (except for gentle dusting with a brush) in order to prevent discontinuous changes in their mass. It was, of course, recognized that checks must be established to ensure the constancy of the mass assigned to the summation of N_1_ and N_2_. Two criteria were routinely used.

**Figure 1:**
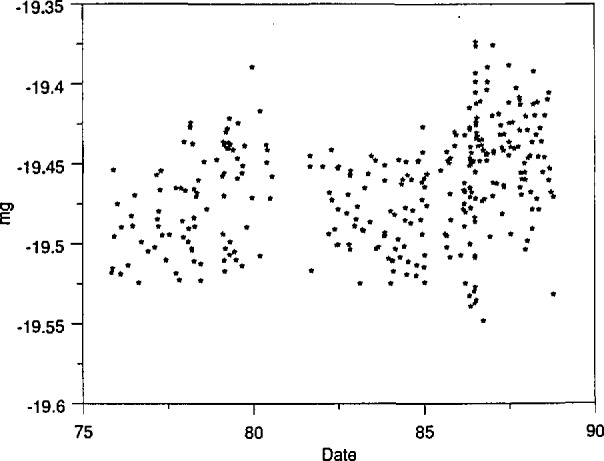
Mass values of N_1_–N_2_ as a function of time. Measurements were taken on a balance having a standard deviation of 0.035 mg for a single reading.

The first criterion was the constancy of *C*. A measurement of *C* was available each time N_1_ and N_2_ were used. In time, a newer balance of similar design was obtained. This device, which is still in use, has a standard deviation of about 0.035 mg. If values of *C* were seen to change significantly with time, it would mean that the summation mass of N_1_ and N_2_ had deviated from its accepted value. This test is effective in checking whether one or the other kilograms has suffered damage since its last use. However, the test fails to detect changes common to both artifacts. Because N_1_ and N_2_ are virtually identical and receive identical use, such changes cannot be ruled out *a priori*. Thus the constancy of *C* is not a sufficient test to rule out a change in the summation mass of the two kilograms. A control chart showing values of *C* over time is given in figure 1. The second criterion is discussed below in section 2.2.2.

In 1969, the masses of N_1_ and of N_2_ were redetermined 10 times with respect to K20 and K4. Measurements were made on a one-pan balance having a standard deviation of 0.14 mg for a single observation. The results of these measurements indicated that N_1_ and N_2_ were an average of 0.09 mg/kg below their accepted value. However, because the uncertainties propagated from the prototype kilograms and from the correction for air buoyancy could still not be assessed, these data were not used.

#### 2.2.2 100-g Check Standards

The second criterion used to monitor the constancy in mass of N_1_ and N_2_ was the evolution in time of two 100-g “check” standards. A measurement of one or the other of these standards in terms of N_1_ and N_2_ was obtained each time a routine calibration was performed on a set of weights from 1 kg to 100 g. Such measurements are carried out dozens of times each year. If the mass of the 100-g check standards was seen to change over time, it would be evidence that either their mass or that of N_1_ and N_2_ was changing. It is unlikely that the mass of the 100-g check standards would change in exact proportion to the mass of the 1-kg working standards. This test suffers, however, from low precision. The statistical precision in the assignment of mass to a 100-g standard is about ten-times lower than the relative precision of mass assigned to 1-kg weights. The reason is simply that all mass comparisons between 1 kg and 100 g are performed on the same balance. One would need to average about 10^2^ mass determinations of a 100-g check standard in order to have the same relative precision as one single mass determination of a 1-kg standard.

**Figure 2:**
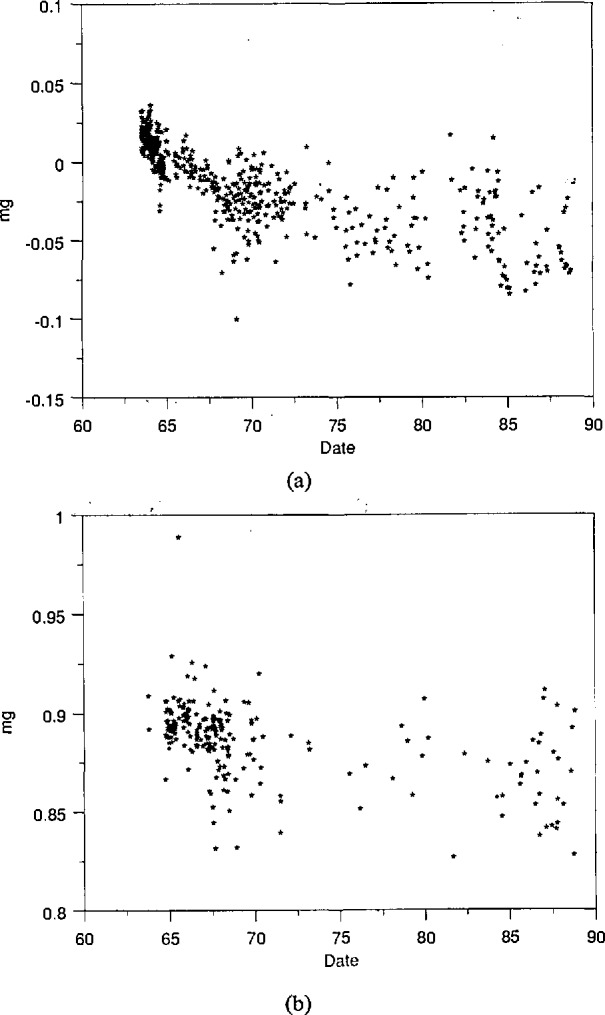
Mass values of 100-g check standards JMC-l(a) and JMC-2(b). These values are based on the accepted mass of N_1_ and N_2_ prior to January 1, 1990.

The 100-g check standard suffers from an additional problem. Since it receives heavy use, its mass can reasonably be expected to decrease with time due to wear. Control charts showing mass values obtained over time for our 100-g check standards, JMC-1 and JMC-2, are given in figure 2. The apparent rapid loss in mass early in the service life of JMC-1 is not unusual. Such behavior is also seen, for instance, in our 1-g check standard where there can be no possibility that the source is instability in 1-kg working standards. Thus the 100-g check standards, while essential to guard against measurement blunders and catastrophic changes in working standards, are themselves susceptible to long-term instability.

#### 2.2.3 State Laboratories

Each state within the United States maintains a well-equipped laboratory for primary mass metrology, typically placed administratively within the State Department of Agriculture. Training of personnel and many aspects of quality control are coordinated through the NIST Office of Weights and Measures (OWM). The OWM organizes regional round-robin measurements involving State mass-standards of various nominal values. These round-robins also include standards recently calibrated by NIST. An examination of round-robin results for 1-kg masses does not reveal systematic differences between NIST and the States developing over time. But the precision of these comparisons limits conclusions to about 0.5 mg/kg.

### 2.3 Fundamental Measurements

Some fundamental constants offer a check on the constancy of mass standards. During the 1970s, measurements of the Avogadro constant *N*_A_ [6] and the Faraday constant *F* relied directly on mass values maintained at NIST. These measurements can be compared with related measurements at other laboratories as is done during periodic CO-DATA adjustments of the fundamental constants [8].

In the case of the NIST determination of the Faraday constant, routine mass calibrations of a 5-g and 3-g working standard were used. It was estimated that the uncertainty in these calibrations was 0.5 ppm (standard error). This estimate contributed less than 10 percent of the combined experimental uncertainty. The Faraday constant has, therefore, little bearing on the present discussion.

This is not true in the case of the Avogadro constant. In order to have their mass values directly traceable to national standards, the experimenters made direct use of K20 and K4. Several calibrations at the 1-kg level were carried out on the newly developed NBS-2 balance [9]. This balance operates under remote control and, at that time, had a standard deviation of less than 0.005 mg. (After initial testing at NBS, the balance was transferred to the BIPM where improved conditions have reduced its standard deviation five-fold.) Unfortunately, N_1_ and N_2_ were not measured during the experiments, although several stainless-steel kilograms were calibrated in terms of K20 and K4. Two of these kilograms had also been measured against K20 and K4 in 1969 as part of the series of mass determinations which included N_1_ and N_2_ (see sec. 2.2.1, above). These results were completely consistent with the 1969 measurements and thus raise the question of whether the mass values for N_1_ and N_2_ dating from 1958 were still appropriate.

## 3. History of Mass Standards after 1980

About 10 years ago, NIST began a program to tie the mass values disseminated by its calibration services with international standards. It was foreseen that improvements in commercial balance technology and improved precision in measuring critical fundamental constants would soon make this step necessary. In addition, questions of international compatibility of national standards began to be raised at this time. In order to assess the presently accepted values of NIST secondary standards with respect to the SI, four major areas had to be addressed:

1. A meaningful calibration of K20 and K4 with respect to accepted representations of SI standards.
2. A reliable method for making corrections for air buoyancy between primary standards of platinum-iridium and secondary standards of nichrome or stainless steel.
3. A balance which could compare kilogram masses with a precision no worse than 0.005 mg.
4. Demonstration that primary standards could indeed be used periodically to calibrate secondary standards and that mass values so determined did not suffer from serious, unexplainable discontinuities.

We now briefly describe efforts made in these four areas.

### 3.1 Tie to International Standards

As mentioned in section 2.2.1 above, the main reason given in the past for not basing mass calibrations on routine comparisons with K20 was that the long-term stability of platinum-iridium prototype kilograms had not been rigorously established. One reason for this apparent lack of understanding is the infrequency with which the International Prototype Kilogram is used. The BIPM faces this same problem because it is their job to recertify national prototype kilograms upon request and to provide new national prototype kilograms when required. These activities must be carried out during the long intervals when the International Prototype Kilogram is not accessible.

As described in [1], the BIPM has set in place the following system in which all the mass standards involved are made of platinum-iridium alloy.

Two working standards are used in the calibration of an unknown prototype. The measured difference in mass between the two working standards is used to check that neither has suffered a catastrophic change in mass. The working standards are cleaned at about 15-year intervals. Within these intervals, however, their mass is redetermined periodically against a third kilogram which is reserved for just this use. This third kilogram is cleaned just prior to its use in recalibrating the working standards. Based on the history of the last 40 years, it appears that the BIPM representation of the SI unit of mass is stable to within about 0.02 mg (0.02 ppm). Therefore, it seems a reasonable goal to achieve compatibility with the mass representation currently maintained at the BIPM. These measurements are reported in detail in [1].

### 3.2 Corrections for Air Buoyancy

In eq (1), the quantity *ρ*_a_ is typically determined from an equation-of-state for moist air. The inputs to this equation are temperature, barometric pressure, relative humidity, and ambient level of carbon dioxide. The last of these has relatively little effect. It is obvious that errors in measuring the required experimental input parameters will propagate to the final result. In the 1970s, however, it was appreciated that the equation-of-state itself has great importance and that several such equations were in wide use. Furthermore, it had not yet been demonstrated experimentally that any of the equations-of-state in use were adequate for actual mass comparisons.

At NIST, Jones derived a semi-empirical equation-of-state based on up-to-date data [10]. This equation, with minor changes, was endorsed for use in mass metrology by the CIPM in 1981 [5]. The equation given in [5] is now referred to as the CIPM-81 equation-of-state for moist air and is used for mass metrology by most national laboratories. The NIST began using this equation for international work in 1981. Use of CIPM-81 instead of its predecessor [11] makes a negligible change to routine mass calibrations. As of January 1, 1990, however, CIPM-81 has been adopted for use in all calibration software.

In order to test the efficacy of CIPM-81, it is necessary to determine the mass difference between two nominally equal weights with and without reliance on the equation-of-state. The latter measurement is typically done in vacuum. This type of comparison was done at the Physikalisch-Technische Bundesanstalt (PTB) [12]. Results agreed to within the expected uncertainty, 1 × 10^−4^ in *ρ*_a_.

It is also necessary to measure the input parameters with sufficient accuracy. In general, this requires the use of transducers whose calibration is checked at frequent intervals by defining instruments. Our capabilities as they existed in 1985 are described in [1]. Since that time, we have improved the accuracy of our measurements of barometric pressure and of relative humidity.

### 3.3 Improved Balance

The balance used for primary mass metrology must operate by remote control in order to ensure that the weights being compared remain in sufficient equilibrium with the air of the weighing chamber. Schoonover and Keller have demonstrated that severe systematic errors may intrude if the equilibrium constraints are violated [13]. In addition, the balance itself must have sufficiently high precision. We consider the balance to be suitable when either of the two following conditions is met: 1. The contribution of the balance imprecision to the uncertainty of working standards is negligible compared to the imprecision of routine mass calibrations. 2. The imprecision of the balance is negligible compared to typical instabilities of mass standards.

In [1], we described modifications made to an existing balance which allowed it to fulfill the first criterion. Although working reasonably well, we wanted to improve efficiency by fully automating it. In order to make the job of automation more straight forward, the balance was fitted with an electro-magnetic servocontrol system [14]. Introduction of the servocontrol also resulted in a modest improvement in precision [15].

### 3.4 Stability of Mass Values

It remains to demonstrate that the work undertaken since 1980 has led to an improved representation of the SI unit of mass.

#### 3.4.1 K20 and K4

**Figure 3:**
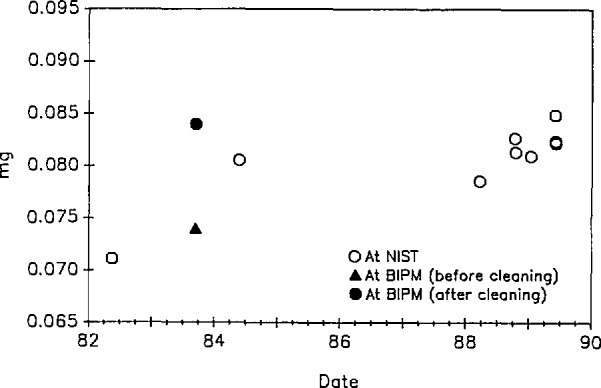
Mass values of K20–K4 as a function of time. Measurements were taken on a balance having a standard deviation of 0.0018 mg for a single reading.

The most recent mass value for kilogram K20 results from the 1984 calibration at the BIPM [1]. As discussed in [1], the cleaning process at the BIPM removed significant amounts of surface pollution from the two prototypes. (The kilograms had also been cleaned at NIST but by a less effective technique). Since 1984, NIST has adopted the BIPM cleaning method. Values obtained for the difference in mass between K20 and K4 are shown in figure 3. These have standard deviation of 0.0019 mg. We would expect a standard deviation of 0.0013 mg based solely on the observed standard deviation of the balance which was used. The difference is negligible.

#### 3.4.2 N_1_ and N_2_

Throughout the last 10 years, N_1_ and N_2_ continued to be used as working standards for routine mass calibrations. In 1982, they were measured against K20 and K4 prior to sending the latter two weights to BIPM for recalibration. The results, calculated after receiving the new BIPM certificate, indicate that the value of *R*/2 was 0.103 mg±0.025 mg below that accepted. The uncertainty is at an estimated level of one standard deviation and is dominated by problems with auxiliary equipment used in measuring air buoyancy. The value of *C* was found to be −19.474 mg±0.003 mg, consistent with the control chart data shown in figure 2.

From 1986 to 1988, mass values of N_1_ and N_2_ were determined three times against K20 and K4 in a more careful series of measurements. Several other stainless-steel kilograms were also involved in the measurements. These are discussed in section 3.4.3, below. It is sufficient to mention at this point that this series of measurements was consistent with the long-term measurements of the other kilograms involved. The results of the 1986–1988 measurements are summarized in table 1. The uncertainty types and the rules for combining uncertainty conform to recommendations of the CIPM [3]. (This reference defines Type A and Type B uncertainties.) Components 2, 4, and 5 will be discussed in more detail in section 3.4.3. In assessing whether the observed change in *R*/2 after 1986 is significant, one must not include Type B components, which we believe to be systematic to all measurements in table 1. It is interesting to note that the observed change in *R*/2 after 1986 is three times greater than the change in *C*. It is also interesting that the data of figure 1 show a statistically significant variation with time. A linear fit to the data predicts that the value of *C* in April 1988 was −19.454+0.0028 mg (1 standard deviation), in satisfactory agreement with the measurement shown in table 1.

During this period, several kilograms which were submitted to NIST for calibration were measured against N_1_ and N_2_ using routine calibration procedures. The test kilograms were also measured against stainless-steel kilograms which are discussed in the next section using our best 1-kg balance. The results were, in all cases, consistent with table 1.

There was now good evidence that the accepted value of *R*/2 was 0.164 mg below the accepted value. Less certain evidence suggests that more than half of this difference had been present since at least 1969 (see sec. 2.2.1). This computes to an average change of order −0.004 ppm/yr.

The standards N_1_ and N_2_ were again checked in 1989. Although these measurements were not as extensive, they show that the average mass had dropped by another 0.05±0.013 mg (1 standard deviation) with respect to four stainless-steel kilograms reserved for special use. This change thus appears to be real and serves as a warning that N_1_ and N_2_ are now losing mass at a greatly increased rate. The value of *C* measured during these measurements had returned to within 0.02 mg of the accepted value.

#### 3.4.3 New Secondary and Working Standards of Mass

Kilograms N_1_ and N_2_ have served as both secondary standards—artifacts of practical density which most accurately represent mass as specified in the SI; and working standards—artifacts of practical density used as standards in routine calibration work. Our intention was to separate these roles by acquisition of new standards, all made of non-magnetic stainless steel. The choice of alloy simply reflects the fact that the highest quality 1-kg weights which are commercially available are now made of stainless steel. Several stainless-steel kilograms were already on hand for use as secondardy standards. Three of these, designated D2, E1, and E2 are about 25 years old. The physical characteristics of all three kilograms are similar; D2 was described in some detail in [1]. We also made use of a newer kilogram, designated CH-1, whose characteristics are also described in [1]. The four artifacts were grouped in pairs: CH-1 and D2 formed one pair while E1 and E2 formed the second pair. When not in use, the pairs were stored in separate containers of different design. The pair E1, E2 was never subjected to any type of cleaning except for gentle dusting with a soft brush. The pair CH-1, D2 was cleaned on various occasions.

**Figure 4:**
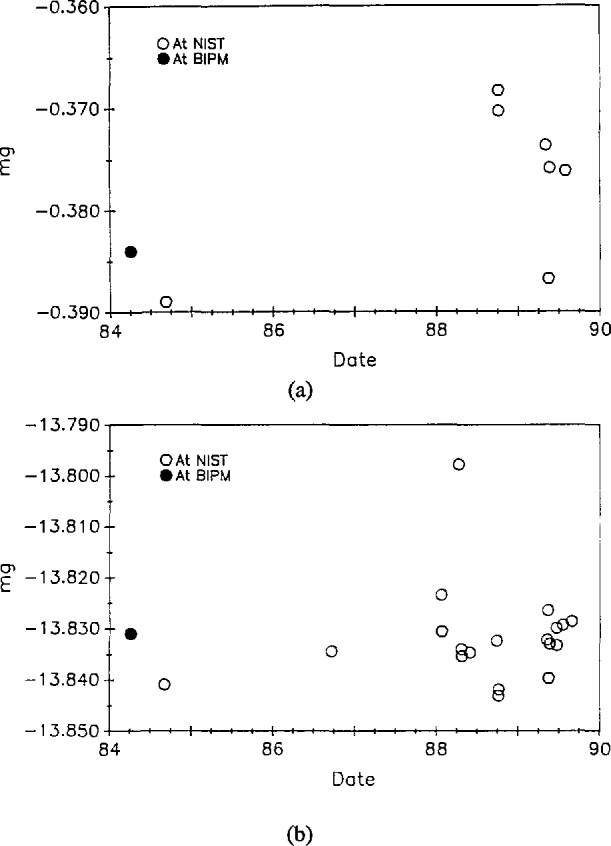
(a) Mass of CH-1 as a function of time. These values are based on direct comparison with K20. The balance used has a standard deviation of 0.0018 mg for a single reading, (b) Mass of CH-1 –D2 as a function of time using the same balance as in (a). There is one outlying point which has been excluded in the data analysis.

**Figure 5:**
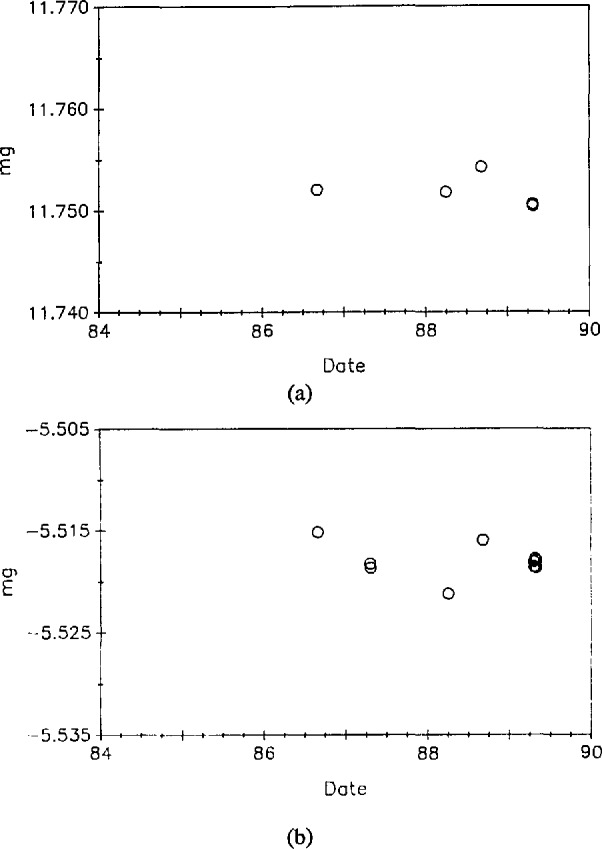
(a) Mass of E1 as a function of time. These values are based on direct comparison with CH-1 and D2. The balance used has a standard deviation of 0.0018 mg for a single reading. (b) Mass of E1 – E2 as a function of time using the same balance as in (a).

The pair CH-1, D2 was compared eight times against primary standards K20 and K4. The mass values of CH-1 resulting from these measurements are shown in figure 4(a). Figure 4(b) shows measurements of the mass difference between CH-1 and D2. Note that results displayed in figure 4(a) include a buoyancy correction of approximately 95 mg while the correction for air buoyancy needed for the results in figure 4(b) was less than 3 mg. Figure 5 shows similar data for the pair E1, E2. In this case, however, the pair CH-1, D2 was used as the standard. The mass value assigned to the standard was the same for all the data shown. Pertinent statistical parameters are summarized in table 2. The outlying point in the mass difference of CH-1 and D2 was repeatable. Because the difference returned to its previous values upon recleaning the two kilograms, we assume the outlying value was due to some type of surface contamination. At any rate, the outlying point is not included in the calculations for table 2.

In table 2, *s*_total_ is the estimated standard deviation of the data shown in figures 4 and 5. The number of degrees of freedom in this estimate is given in the next column. The quantity *s*_w_ refers to the “within-group” standard deviation—that component of the observed standard deviation which can be attributed to the balance precision. This number is pooled from a great many measurements and thus has a large number of degrees of freedom. The “between-group” standard deviation, *s*_b_, is a measure of increased variability seen over long time periods. This quantity is calculated from the others in the table. The estimated number of degrees of freedom [16] in *s*_b_ is given in the last column. A full discussion of these parameters as well as their treatment in the context of mass calibrations has been given by Croarkin [17]. It is interesting to note that the data of figure 2, when subjected to the same analysis, indicate that *s*_b_ for these measurements is 0.0116 mg [17].

The Croarkin model is not sufficient to model direct comparisons of CH-1 and D2 with K20 and K4. This is because uncertainties in buoyancy corrections have little effect on measured differences between weights of the same density but have large effects on measured differences between weights of different density. While the transducers used to measure the parameters of temperature, pressure, relative humidity, and carbon-dioxide level have excellent short-term precision, slow drifting between recalibration leads to an additional “between group” uncertainty. If the error model of [17] is extended to include buoyancy effects, the data of table 2 can be used to compute an additional parameter *s_ρ_* = 0.007 mg (DF=3.9). This parameter characterizes daily variability in the measured mass difference between a kilogram of platinum-iridium and a kilogram of stainless steel due solely to measurement of the air buoyancy correction.

Although based on somewhat limited data, it seems that E1 and E2, kilograms of the identical alloy and which are never cleaned, have a more stable mass than CH-1 and D2. This is a curious result in the sense that mass values for E1 and E2 are based on direct comparison with CH-1 and D2. In these comparisons, it is assumed that the summation mass of CH-1 and D2 is the average of all recent measurements which are in statistical control. The evidence thus suggests that this average is a better estimate of the mass of CH-1 and D2 than, for instance, the most recently obtained values.

As mentioned in the introduction to this section, it was envisioned that use of N_1_ and N_2_ as working standards would be superseded by stainless-steel kilograms. These would have a nominal density of 8000 kg·m^−3^. In 1985, six such kilograms, identical to CH-1, were obtained for this purpose. They are marked 1,2,…,6 but for purposes of discussion we shall refer to them as C1, C2,…,C6. Until January 1988, these six kilograms were used extensively for various cleaning studies. Now, however, they will be used as working standards as described below in section 4.

## 4. Summary of the Change on January 1, 1990

Beginning on January 1, 1990, the mass values assigned to working standards of the NIST calibration service are based on a calibration chain which starts with mass values assigned to NIST primary standards K20 and K4 by the BIPM, continues with mass values assigned to secondary standards CH-1 and D2 with direct reference to K20 and K4, and finally to working standards C1, C2,…,C6 by direct reference to CH-1 and D2.

### 4.1 Effect on Industry and Technology

An Ad Hoc Committee of the National Conference of Standards Laboratories (NCSL) was formed in order to help assess industrial and technological implications of the actions contemplated for January 1, 1990. Members of the Committee include representatives from civilian and military standards laboratories, balance manufacturers, and weight manufacturers. All were asked to estimate the impact which a change of roughly 0.15 mg/kg would have on their programs. The members could not identify a single instance where such a change would affect a manufactured product or a critical measurement. Virtually all concerned, however, recognized that a change of this magnitude could be noticeable within their metrology laboratory. This is not surprising since typical NIST calibrations give an uncertainty of about 0.075 mg (3 standard deviations) for calibrations of 1-kg standards and users of these standards often have balances of comparable precision to our own.

In recent years, calibrations for primary national laboratories of other countries have been carried out using secondary standards CH-1 and D2 with assigned values based directly on measurements against K20. These measurements are not, therefore, in need of correction.

### 4.2 Implementation

Based on the data shown in section 3.4.2, it is clear that, by 1988, mass values assigned to NIST working standards were some 0.164 mg/kg higher than our best estimate of their actual value (that is, the value directly traceable to the representation of the SI unit of mass). At the beginning of the decade, the discrepancy was about 0.10 mg/kg. There is evidence that, between 1988 and 1989, the discrepancy grew still greater.

In early 1988, and based on the data available to that point, it was decided to assign new mass values to NIST working standards on January 1, 1990. On the same date, the new quality-control procedures designed to keep mass values assigned to NIST working standards closely tied to the SI representation of mass would be in place. Various standards organizations were informed of these intentions by letter. The letter also stated that the new mass values would be of the order of 0.15 ppm lower than the present values. Also in 1988, the NCSL Ad Hoc Committee was established to help in the implementation of the change. The target date of January 1, 1990 was chosen to coincide with the date on which international changes in the representations of the SI volt, ohm, and kelvin would be implemented. Guidelines developed by the Ad Hoc Committee are given in the Appendix.

These guidelines treat the discrepancy between the accepted mass of NIST working standards and the mass traceable to SI representations as equal in magnitude to 0.17 mg/kg (0.17 ppm) throughout the decade from 1980 through 1989. Based on data presented above, we see that this is an oversimplification. Our best data, taken between 1986 and 1988, give the discrepancy as 0.164 mg/kg. Less accurate data, however, suggest that the discrepancy grew slowly throughout the decade and then increased rapidly in the last year. A time-dependent correction algorithm with time-dependent uncertainty could, of course, be devised based on these data. The complexity of applying such an algorithm combined with its trivial scientific or technological benefit made this course unwise. Instead we recommend correction of −0.17 mg/kg made to NIST calibration certificates dated during the 1980s. This, we believe, will provide sufficient continuity with certificates issued after January 1, 1990.

The BIPM is conducting the 3rd verification of national prototype kilograms. When this exercise is completed (perhaps in 2 years) we will have a much better idea of the internal stability of BIPM standards and the stability of these standards with respect to the national prototype kilograms. For the present, we estimate that the mass values used by NIST in its calibrations represent SI values as maintained by the BIPM to within 0.03 mg/kg or 0.03 ppm (1 standard deviation). This uncertainty will not be included in NIST calibration reports except to say that it is systematic to all mass measurements.

## 5. Future Plans

We plan to participate in the 3rd verification of national prototype kilograms being organized by the BIPM. Consequently, in early 1990, we will send our national prototype (K20) to BIPM for a lengthy set of comparisons.

We plan to recalibrate our working standards in terms of secondary standards CH-1 and D2 at approximately 6-month intervals. The working standards will not, initially, be cleaned although the secondary standards will. We foresee calibrating the secondary standards in terms of our primary standards K20 and K4 at about 2-year intervals. Based on the data presented above, we believe this procedure will permit us to know the mass ratio between our working standards and our primary standards to within 0.01 ppm (1 standard deviation) at all times. As noted at the end of the previous section, this uncertainty does not include possible discrepancies between NIST standards and those of the BIPM. We tentatively set the latter uncertainty at 0.03 ppm (1 standard deviation).

It would be helpful to have a balance of 1-kg capacity and a standard deviation of order 0.005 mg for use in routine calibration work. Such a device would help compensate for the fact that, since January 1, 1990, we are formally recognizing that our working standards are subject to uncertainty.

A major goal of the new quality-control system is to improve international compatibility regarding practical mass standards. We are, therefore, seeking to promote international comparisons of stainless-steel mass standards in order to ascertain the degree of compatibility among various industrialized countries.

In conclusion, we note that a system of metrology ultimately based on an artifact standard will necessarily have shortcomings. Over a long enough period of time, mass differences between any two artifact standards will be unstable; the estimated standard deviation based on the complete data record will diverge. If the mass of one of the artifacts is arbitrarily assumed to be constant, its actual instability will in time be revealed by measurements of true physical constants. While there has as yet been no such revelation [18], modern technology may soon be expected to put the present definition of the SI kilogram to a severe test.

## Figures and Tables

**Table 1:** Recent determinations of the masses of kilograms N_1_ and N_2_ with respect to secondary standards calibrated against K20. The values of C are subject to a measurement uncertainty of 0.0013 mg (1 standard deviation)

Date	*R*/2 [(N_1_ + N_2_)/2]	*C* [N_1_−N_2_]
1986 Aug	1 kg −5.159 mg	−19.440 mg
1987 Nov	1 kg −5.192 mg	−19.451 mg
1988 Apr	1 kg −5.193 mg	−19.447 mg
Accepted values:	1 kg −5.0295 mg	−19.476 mg (1965)
		−19.454 mg (Apr. 1988)
Uncertainty (1 standard deviation or 68 percent confidence level) for measured values of *R*/2		

Component		Type A	Type B
1.	Instability of K.20 since 1985 BIPM calibration	included in 4	nil
2.	Calibration precision of secondary standard	0.0035 mg	nil
3.	Correction of secondary standards for air buoyancy	included in 2	0.01 mg
4.	Instability of secondary standards	0.0036 mg	nil
5.	Calibration precision of (N_1_ + N_2_)/2	0.0005 mg	nil
6.	Correction of N_1_ and N_2_ for air buoyancy	nil	0.001 mg
	RSS	0.0050 mg	0.010 mg
Combined Type A and B: 0.011 mg			

**Table 2:** Statistical parameters inferred from measurements of secondary standards

Mass of:	*^S^*total	DF	*S*_w_	*S*_b_	DF
CH-1	0.0085 mg	8	0.0013 mg		
CH-1-D2	0.0052 mg	18	0.0013 mg	0.0036 mg	16.9
E1	0.0015 mg	4	0.0011 mg	0.0011 mg	2.5
E1-E2	0.0016 mg	9	0.0013 mg	0.0009 mg	4.2

## Footnotes

1 1 ppm = 1 part per million = 1 × 10^−6^.

## 6. Appendix. Notice of Change in the Unit of Mass Traceable to The National Institute of Standards and Technology

On January 1, 1990 the unit of mass as disseminated by the National Institute of Standards and Technology (NIST) will shift by 0.17 mg/kg (0.17 ppm). This small shift will bring the unit of mass traceable to NIST into better agreement with international standards. Since the avoirdupois pound is defined as 0.45359237 kg, pound masses traceable to NIST will also be affected to the same extent (0.17 *μ*1b/1b, or 0.17 ppm).

Most people will be unaffected by this small change so that continued traceability to NIST can be maintained without taking any action. Unaffected users will be those whose mass standards are assigned an uncertainty greater than 1 mg/kg or 1*μ*1b/1b (1 ppm). Included in *un* affected list are:

1. Analytical weights certified to be within any of the tolerances prescribed by NIST/NBS or ASTM/ANSI or to any OIML tolerance except E_1_.
2. Direct-reading balances and scales.
3. Any analytical weights which have been assigned an uncertainty greater than 1 mg/kg or 1 *μ*1b/1b (1 ppm). [This will typically include all weights greater than 2 kg or less than 20 g which were calibrated by NIST/NBS (see table A1). In some special cases, however, NIST calibrations at weight denominations other than those shown in table A1 may have an uncertainty lower than 1 mg/kg.]

Traceability to NIST of the above three categories is unaffected by the change which will take effect on January 1, 1990. No action need be taken. In addition, any calibration certificate dated January 1, 1990 or later already has any necessary changes incorporated.

**Table A1:** Typically, action need be taken only for these nominal values of weights *and* only if the assigned uncertainty is below the value given. Although this table shows weight denominations most likely to require correction, denominations which may require correction are not necessarily limited to those shown

Nominal mass	Uncertainty	Nominal mass	Uncertainty
2 kg	2.00mg	50 lb	50 *μ*lb
1 kg	1.00 mg	30 lb	30 *μ*lb
		20 lb	20 *μ*lb
500 g	0.50 mg	10 lb	10 *μ*lb
300 g	0.30 mg		
200 g	0.20 mg	5 lb	5 *μ*lb
100 g	0.10 mg	3 lb	3 *μ*lb
		2 lb	2 *μ*lb
50 g	0.05 mg	1 lb	1 *μ*lb
30 g	0.03 mg		
20 g	0.02 mg	0.5 lb	0.5 *μ*lb
		0.3 lb	0.3 *μ*lb
		0.2 lb	0.2 *μ*lb

Weights which will be affected by the change which will take effect on January 1, 1990 are all those which do not fall into category 3 above and, in addition, whose calibration certificate bears a date before January 1, 1990. Affected weights are those which have an assigned calibration uncertainty of less than 1 mg/kg (1 ppm). Based on typical NIST calibration reports, these will generally be weights with denominations between 2 kg and 20 g or 5 lb and 0.2 lb. Other denominations may be affected in special cases, however.

The following actions will be necessary in order to maintain traceability to NIST for the affected weights:

- Weights whose calibration certificate bears a date after January 1, 1980 and before January 1, 1990.
  After January 1, 1990 the mass of each affected weight should be *reduced* by 0.17 mg/kg (0.17 ppm) as shown in table A2. This applies both to the true mass and the apparent mass. The uncertainty stated in the report remains the same.
  (Alternatively, the mass values stated in the calibration certificate may remain uncorrected provided the stated uncertainty is increased by 0.17 mg/kg).
- Weight sets whose calibration certificate bears a date before January 1, 1980 but which have been subjected to a surveillance test within the 10 years preceding January 1, 1990. (An example of a surveillance report issued by NIST is shown at the end of this Appendix.
  After January 1, 1990 the mass of each affected weight should be *reduced* by 0.17 mg/kg (0.17 ppm) as shown in table A2. This applies both to the true mass and the apparent mass. The assertions of the surveillance report will remain in effect.
  (Alternatively, the mass values stated in the calibration certificate may remain uncorrected provided the stated uncertainty is increased by 0.17 mg/kg).
- Weights whose calibration certificate bears a date before January 1, 1980 and which have had no surveillance test subsequent to January 1, 1980.
  After January 1, 1990 the uncertainty assigned to each affected weight should be increased by 0.17 mg/kg (0.17 ppm) until a new calibration or surveillance test is performed.

**Table A2:** Corrections to apply to calibrations dated between January 1, 1980 and January 1, 1990. The denominations shown are those of table A1

Nominal mass	Correction	Nominal mass	Correction
2 kg	−0.3400 mg	50 lb	−8.500 *μ*lb
1 kg	−0.1700 mg	30 lb	−5.100 *μ*lb
		20 lb	−3.400 *μ*lb
500 g	−0.0850 mg	10 lb	−1.700 *μ*lb
300 g	−0.0510 mg		
200 g	−0.0340 mg	5 lb	−0.850 *μ*lb
100 g	−0.0170 mg	3 lb	−0.510 *μ*lb
		2 1b	−0.340 *μ*lb
50 g	−0.0085 mg	1 lb	−0.170 *μ*lb
30 g	−0.0051 mg		
20 g	−0.0034 mg	0.5 lb	−0.085 *μ*lb
		0.3 lb	−0.051 *μ*lb
		0.2 lb	−0.034 *μ*lb

### Sample Surveillance Report Issued by NIST

April 1, 1987

In reply refer to: 731/12345

Company XYZ

1 Metrology Blvd.

Grovers Corner, NJ 00000

Attention: J. Doe

Subject: Recalibration of Mass Standards previously calibrated under NBS Test No. 00/G00000 (copy attached)

Items: Nine (9) Mass Standards: 100 g–1 g

The above items have been intercompared in sums. The differences as measured have been compared with the differences computed from the value under G00000. One or more of the items have been checked against national standards. The results of this test indicate that there is no significant change since the last calibration. This test assures the continuing accuracy of the values under G00000.

Sincerely,

Richard S. Davis

Group Leader, Mass Group

Center for Manufacturing Engineering

Attachment
